# Tracking Orangutans from the Sky

**DOI:** 10.1371/journal.pbio.0030022

**Published:** 2004-12-07

After apprenticing at her mother's side for some eight years—the first three clinging to her body—an orangutan is ready to make her own way in the forest canopy. The only great ape specializing in arboreal living, orangutans forage the treetops mostly for fruit, nuts, insects, leaves, and tree bark. They can recognize hundreds of species of edible fruit from trees and woody climbers and remember their location and fruiting season. Malay legend says orangutans (a variation on the Malay trope for “man of the woods”) derived from humans who sought refuge from their species in the wilds of the forest. Today, with the last orangutan refuges shrinking drastically, the man of the woods has nowhere else to go.

Hundreds of thousands of orangutans once ranged throughout southeast Asia. Now just two orangutan species inhabit just two countries: Indonesia (Kalimantan, the southern part of Borneo and Sumatra Island) and Malaysia (Sabah and Sarawak, in northern Borneo). The Sumatran orangutan is listed as critically endangered; the Bornean, endangered. In Indonesian Borneo and Sumatra, logging operations clear an estimated 5 to 6 million forest acres a year, leaving the apes stranded in isolated stands of trees and the normally fire-resistant rainforest at sudden risk. Another force driving orangutan extinction in Indonesia is the poaching and illegal killing (mothers do not give up their babies without a fight) that secures orangutan babies for the exotic pet trade. In Sabah, Malaysia, the primary threat comes from clearing the forest for agriculture.

Conservation efforts depend, among other things, on having reliable data on population size, density, and distribution, but estimates of orangutan numbers in Sabah—which range from 2,000 to 20,000—are outdated. In a new study, Marc Ancrenaz and colleagues report an innovative method of directly estimating orangutan numbers from the number of nests detected during aerial surveys. (Orangutans are tough to spot directly, so researchers count the nests they sleep in at night.) Their survey, which covered the entire orangutan range throughout Sabah, estimates a total population of 11,000 orangutans—a drop of 35% in the past 20 years, based on a 1988 World Wildlife Federation report.

Counting orangutans from the ground can be very time-consuming, difficult work, especially when faced with the hip-deep muck and steep slopes of the rainforest floor. Though helicopters obviously cover greater distance and more remote territory than is possible by foot, they're generally used to survey animals in more open landscapes. By using ground survey data to refine their aerial survey results, Ancrenaz and colleagues could directly assess the distribution and size of orangutan populations throughout Sabah. (Sabah covers roughly 72,000 square kilometers.)

Over the course of two years, ground surveys—requiring 1,100 hours of field work—and aerial surveys—requiring just 72 hours—were conducted throughout all the major forests of Sabah. Commercial logging occurs in about 76% of all Sabah forests in commercial forest reserves. During the overflights, information was recorded on altitude, forest type, forest disturbance (on a scale from none to active exploitation), and signs of human activity.

The authors attribute the 35% decline in Sabah orangutan numbers primarily to habitat loss from agricultural development. The surveys revealed that lowland forests harbored the greatest density of nests and orangutans, with the densest populations found in several highly disturbed, fragmented forests along newly created palm oil plantations. These high orangutan densities could reflect an influx of refugees from recently destroyed forest habitat into areas that are still forested. In logged forests, higher population densities were found in old exploited or sustainably logged forests than in conventionally logged reserves.

While the authors acknowledge the density estimates could be more precise—better measures of nest decay and construction rates are needed—their survey reveals crucial information on orangutan numbers and distribution. Most orangutans in Sabah, including those making up one of the largest unfragmented populations in Borneo, live outside protected areas, in commercially exploited forests. These results suggest that orangutans may adapt better to degraded forests than previously thought—provided illegal hunting and agricultural conversion are controlled.

More field research will help quantify the impacts of human activity—from logging to stealing babies—on great ape ecology and survival, and determine whether exploited forests can support conservation. It may be, for example, that integrating agricultural fields with forested corridors could sustain orangutan populations over the long term. With time of the essence, these aerial surveys will speed that work, and help sustain orangutans' refuge in the treetops, above their human relatives.

